# MicroRNA 130a Regulates both Hepatitis C Virus and Hepatitis B Virus Replication through a Central Metabolic Pathway

**DOI:** 10.1128/JVI.02009-17

**Published:** 2018-03-14

**Authors:** Xiaoqiong Duan, Shilin Li, Jacinta A. Holmes, Zeng Tu, Yujia Li, Dachuan Cai, Xiao Liu, Wenting Li, Chunhui Yang, Baihai Jiao, Esperance A. Schaefer, Dahlene N. Fusco, Shadi Salloum, Limin Chen, Wenyu Lin, Raymond T. Chung

**Affiliations:** a Institute of Blood Transfusion, Chinese Academy of Medical Sciences and Peking Union Medical College, Chengdu, China; b Liver Center, Department of Medicine, Massachusetts General Hospital, Harvard Medical School, Boston, Massachusetts, USA; c Gastrointestinal Division, Department of Medicine, Massachusetts General Hospital, Harvard Medical School, Boston, Massachusetts, USA; d Department of Microbiology, College of Basic Medical Science, Chongqing Medical University, Chongqing, China; e Department of Infectious Disease, The Second Affiliated Hospital, Chongqing Medical University, Chongqing, China; f Department of Infectious Disease, Anhui Provincial Hospital, Anhui Medical University, Hefei, China; Hudson Institute of Medical Research

**Keywords:** PKLR, hepatitis B virus, hepatitis C virus, microRNA, pyruvate

## Abstract

Hepatitis C virus (HCV) infection has been shown to regulate microRNA 130a (miR-130a) in patient biopsy specimens and in cultured cells. We sought to identify miR-130a target genes and to explore the mechanisms by which miR-130a regulates HCV and hepatitis B virus (HBV) replication. We used bioinformatics software, including miRanda, TargetScan, PITA, and RNAhybrid, to predict potential miR-130a target genes. miR-130a and its target genes were overexpressed or were knocked down by use of small interfering RNA (siRNA) or clustered regularly interspaced short palindromic repeat (CRISPR)/Cas9 guide RNA (gRNA). Selected gene mRNAs and their proteins, together with HCV replication in OR6 cells, HCV JFH1-infected Huh7.5.1 cells, and HCV JFH1-infected primary human hepatocytes (PHHs) and HBV replication in HepAD38 cells, HBV-infected NTCP-Huh7.5.1 cells, and HBV-infected PHHs, were measured by quantitative reverse transcription-PCR (qRT-PCR) and Western blotting, respectively. We selected 116 predicted target genes whose expression was related to viral pathogenesis or immunity for qPCR validation. Of these, the gene encoding pyruvate kinase in liver and red blood cell (PKLR) was confirmed to be regulated by miR-130a overexpression. miR-130a overexpression (via a mimic) knocked down PKLR mRNA and protein levels. A miR-130a inhibitor and gRNA increased PKLR expression, HCV replication, and HBV replication, while miR-130a gRNA and PKLR overexpression increased HCV and HBV replication. Supplemental pyruvate increased HCV and HBV replication and rescued the inhibition of HCV and HBV replication by the miR-130a mimic and PKLR knockdown. We concluded that miR-130a regulates HCV and HBV replication through its targeting of PKLR and subsequent pyruvate production. Our data provide novel insights into key metabolic enzymatic pathway steps regulated by miR-130a, including the steps involving PKLR and pyruvate, which are subverted by HCV and HBV replication.

**IMPORTANCE** We identified that miR-130a regulates the target gene *PKLR* and its subsequent effect on pyruvate production. Pyruvate is a key intermediate in several metabolic pathways, and we identified that pyruvate plays a key role in regulation of HCV and HBV replication. This previously unrecognized, miRNA-regulated antiviral mechanism has implications for the development of host-directed strategies to interrupt the viral life cycle and prevent establishment of persistent infection for HCV, HBV, and potentially other viral infections.

## INTRODUCTION

MicroRNAs (miRNAs) are a class of noncoding small RNA molecules (about 22 nucleotides) which regulate gene expression through RNA silencing and posttranscriptional regulation (1). Growing evidence demonstrates that miRNAs play an important role in the regulation of metabolism, viral infection, and host immunity (2–12). miRNAs have been reported as a new class of regulators of metabolism as well as viral infection (4, 8–10, 12–14).

Immunity and metabolism play pivotal roles in maintaining human homeostasis and host defenses, especially in response to external stimuli, such as viral infection (15–19). The energy metabolism of host cells is important for both host and virus, as viruses strictly depend on the host's energy and biosynthetic pathways for their replication and morphogenesis. Pyruvate (also known as pyruvic acid) is a key intermediate in several metabolic pathways. It is generated from glucose through glycolysis, where the phosphoenolpyruvate (PEP) step is catalyzed by pyruvate kinase (PK) (20). There are four tissue-specific isozymes of PK in vertebrates. The L (liver) and R (erythrocytes) isozymes are encoded by the same gene (*PKLR*). Pyruvate kinase isoenzyme L (PKL) is expressed primarily in hepatocytes.

Hepatitis C virus (HCV) chronically infects over 71 million people and hepatitis B virus (HBV) infects over 240 million people worldwide, and these viruses are the leading causes of chronic viral liver disease (21–24). HCV and HBV share many common characteristics despite their distinctly different genome structures. Both HBV and HCV infections induce myriad host changes, including metabolic changes, which may lead to the development of liver cirrhosis and hepatocellular carcinoma (HCC) (14, 25–29). However, the mechanisms by which miR-130a, HCV, HBV, PKLR, and pyruvate metabolism are interconnected remain elusive. We hypothesized that miR-130a regulates HCV and HBV replication through pyruvate metabolism.

The aims of our study were to identify miR-130a target genes and to define the molecular mechanism by which miR-130a regulates HCV and HBV replication. Using bioinformatics tools, we identified that miR-130a regulates *PKLR*. Further, we found that miR-130a regulates both HCV and HBV replication in addition to *PKLR*. We explored the regulatory effect of miR-130a on HCV replication in HCV JFH1-infected Huh7.5.1 cells and primary human hepatocytes (PHHs) and on HBV replication in HepAD38 cells (30) and HBV infectious supernatant-infected, sodium taurocholate-cotransporting polypeptide (NTCP)-transfected Huh7.5.1 cells or PHHs (31). Clustered regularly interspaced short palindromic repeat (CRISPR)/Cas9 gene editing is a powerful, efficient, and reliable means of knocking down a target gene (32, 33). We assessed the effects of a series of target genes on HCV and HBV replication by using CRISPR guide RNA (gRNA), small interfering RNA (siRNA), miRNA mimic, miRNA inhibitor, and overexpression systems. We confirmed that the *PKLR* gene is a direct target of miR-130a. We clarified that miR-130a regulates HCV and HBV replication as well as *PKLR*, and therefore (likely) pyruvate metabolism. Our findings indicate that the metabolic enzymatic step controlled by *PKLR*, which results in pyruvate production, plays an important role in HCV and HBV infections. This previously unrecognized miRNA-regulated antiviral mechanism has implications for the development of host-directed strategies to interrupt the viral life cycle and prevent establishment of persistent infection for HCV, HBV, and potentially other viral infections.

## RESULTS

### Bioinformatic prediction of miR-130a targets.

We applied four prediction algorithms, miRanda, RNAhybrid, TargetScan, and PITA, and obtained 2,152 potential target genes for miR-130a (https://drive.google.com/open?id=0B5t4LtawAMViZ1VxTHlHaWRnblE). We identified 534 genes that were predicted by more than two algorithms, among which 116 genes were related to antiviral responses or liver disease, based on prior publications on PCR and characterization (https://drive.google.com/open?id=0B5t4LtawAMViZ1VxTHlHaWRnblE). These genes were then validated by quantitative PCR (qPCR), and we found that the expression of three genes, encoding pyruvate kinase in liver and red cell (*PKLR*), interleukin-18 binding protein (*IL18BP*), and the low-density lipoprotein receptor (*LDLR*), was significantly downregulated by overexpression of miR-130a (by use of a miR-130a mimic) compared to that with the negative-control mimic treatment (data not shown).

### miR-130a targets *PKLR*.

**FIG 1:**
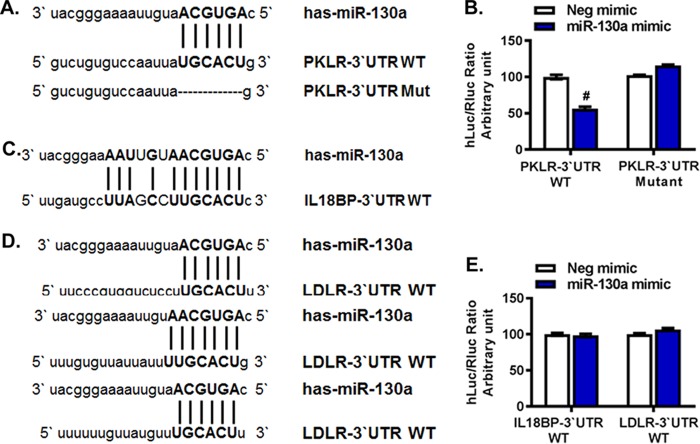
miR-130a directly targeted the *PKLR* 3′ UTR. Huh7.5.1 cells were cotransfected with a cloned pEZX-MT06 plasmid (containing PKLR-3′UTR-WT, PKLR-3′UTR-Mut, IL18BP-3′UTR-WT, or LDLR-3′UTR-WT) and a miR-130a mimic or negative-control (Neg) mimic. The hLuc/Rluc ratio serves as a measure of the inhibition of luciferase expression due to the binding of miR-130a with each cloned 3′ UTR. Each experiment was repeated in triplicate. #, *P* < 0.001 compared to negative-control miRNA. (A) Putative binding sites of the miR-130a seed sequence on PKLR-3′UTR-WT. PKLR-3′UTR-Mut was constructed by deleting the matching base sequence. (B) The miR-130a mimic significantly reduced *PKLR* signaling. The reduction of the hLuc/Rluc ratio disappeared when the binding sites on *PKLR* were deleted. (C) Putative binding sites of the miR-130a seed sequence on IL18BP-3′UTR-WT. (D) Sequences of three putative binding sites of miR-130a on the *LDLR* 3′ UTR. (E) The miR-130a mimic did not affect *IL18BP* or *LDLR* signaling.

To determine whether the *PKLR*, *IL18BP*, and *LDLR* genes are direct targets of miR-130a, we performed dual-luciferase mutation assays using the miR-130a (AGUGCAA) seed sequence as the binding sequence. We identified one potential binding site each in the 3′ untranslated regions (UTRs) of *PKLR* and *IL18BP* and three possible binding sites in the 3′ UTR of *LDLR* (Fig. 1). The 3′ UTRs of *PKLR*, *IL18BP*, and *LDLR* were cloned downstream of the firefly luciferase (hLuc) reporter gene in the reporter vector pEZX-MT06, which was cotransfected with the miR-130a mimic or a negative-control mimic into Huh7.5.1 cells. We found that overexpression of miR-130a significantly repressed the hLuc/Renilla luciferase (Rluc) ratio (by 45%) when miR-130a was cotransfected with the wild-type *PKLR* 3′ UTR (PKLR-3′UTR-WT) compared to that with the negative-control mimic (Fig. 1B). In contrast, no significant changes were observed when miR-130a was cotransfected with an IL18BP-3′UTR-WT or LDLR-3′UTR-WT plasmid (Fig. 1C to E). These results indicate that *PKLR*, not *IL18BP* or *LDLR*, is the target gene of miR-130a. To confirm the direct binding between the 3′ UTR of *PKLR* and the miR-130a seed sequence, we mutated the 3′ UTR of *PKLR* by deleting the binding site of miR-130a. We found that the hLuc/Rluc ratio in the dual-luciferase reporter assay was rescued after deleting the binding sites of miR-130a (Fig. 1B). These results confirm that miR-130a directly targets the 3′ UTR of *PKLR*.

### miR-130a regulates HCV replication through targeting of *PKLR*.

**FIG 2:**
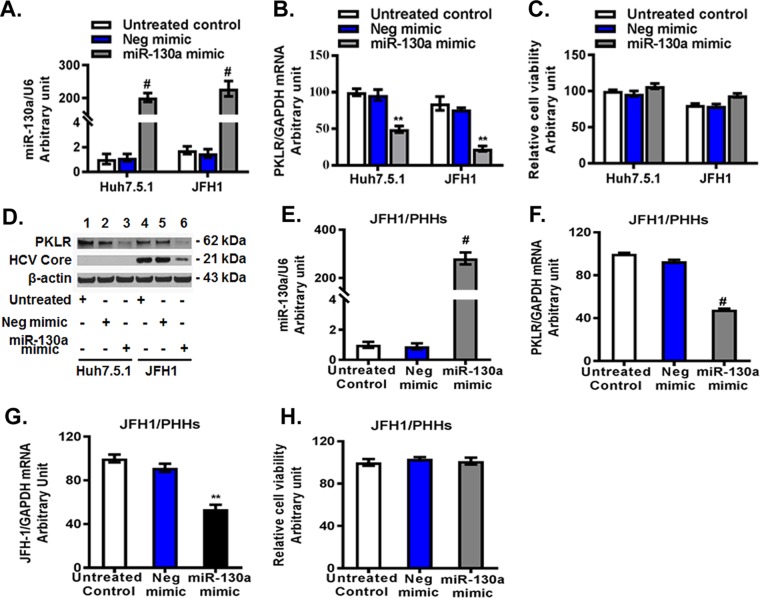
Overexpression of miR-130a inhibited *PKLR* expression and HCV replication. The negative-control mimic or miR-130a mimic was transfected into Huh7.5.1 cells or primary human hepatocytes (PHHs). HCV JFH1 was inoculated into the appropriate wells. miR-130a, HCV RNA, and *PKLR* mRNA levels were tested by qPCR. The miR-130a level was normalized to the U6 level, and other selected gene mRNA levels were normalized to the *GAPDH* mRNA level, yielding arbitrary units (fold changes). Protein lysates were harvested for Western blotting. *, *P* < 0.05; **, *P* < 0.01; #, *P* < 0.001 (for comparisons of the indicated miR-130a and negative-control mimic treatments without or with HCV JFH1 infection). (A) The miR-130a mimic increased the miR-130a level. (B) The miR-130a mimic reduced the *PKLR* mRNA level. (C) The miR-130a mimic did not affect cell viability. (D) The miR-130a mimic significantly decreased PKLR and HCV core protein levels. (E) The miR-130a mimic significantly increased the miR-130a mRNA level in PHHs. (F) The miR-130a mimic significantly reduced the *PKLR* mRNA level in PHHs. (G) The miR-130a mimic significantly inhibited the HCV RNA level in JFH1-infected PHHs. (H) The miR-130a mimic did not affect cell viability in PHHs.

**FIG 3:**
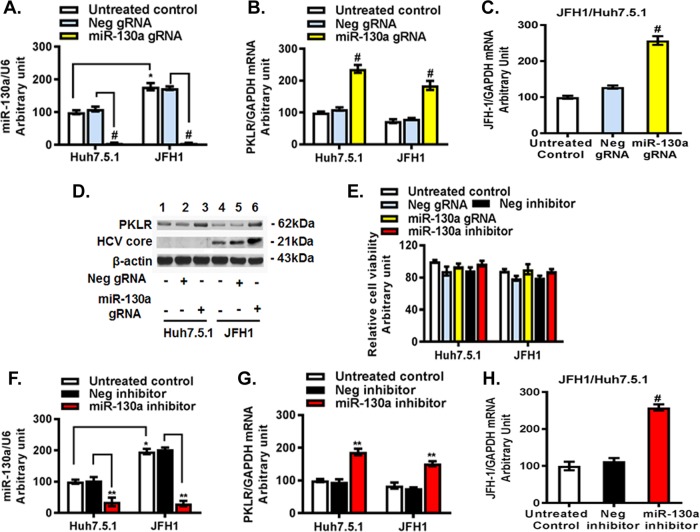
miR-130a gRNA and a miR-130a inhibitor increased *PKLR* expression and HCV replication. A miR-130a hairpin inhibitor or CRISPR/Cas9 miR-130a gRNA was used to knock down miR-130a expression. The miR-130a inhibitor, negative-control inhibitor, miR-130a gRNA, and negative-control gRNA were transfected into Huh7.5.1 cells. HCV JFH1 was inoculated into the appropriate wells and incubated for 48 h. (A) miR-130a gRNA decreased the miR-130a level. (B) miR-130a gRNA increased the *PKLR* mRNA level. (C) miR-130a gRNA promoted HCV RNA replication. (D) miR-130a gRNA increased PKLR and HCV core protein levels. (E) miR-130a gRNA or the miR-130a inhibitor did not affect cell viability. (F) The miR-130a inhibitor decreased the miR-130a level. (G) The miR-130a inhibitor increased the *PKLR* mRNA level. (H) The miR-130a inhibitor promoted HCV RNA replication.

miR-130a expression has been reported to be upregulated in liver tissues of HCV-infected patients (34). We observed an approximately 2-fold increase of miR-130a in JFH1-infected Huh7.5.1 cells compared to that in uninfected cells (Fig. 2A). We sought to investigate the association of *PKLR* with miR-130a and HCV replication. We found that overexpression of miR-130a led to *PKLR* reductions at both the mRNA and protein levels in Huh7.5.1 cells (Fig. 2A to D). The miR-130a mimic did not significantly affect the viability of Huh7.5.1 cells (Fig. 2C). We also observed a significant decrease in HCV core protein in JFH1-infected Huh7.5.1 cells after miR-130a overexpression (Fig. 2D). Finally, we confirmed the inhibitory effect of miR-130a overexpression on *PKLR* mRNA (Fig. 2F) and HCV RNA (Fig. 2G) expression in JFH1-infected PHHs (Fig. 2E to H). The miR-130a mimic did not significantly affect the viability of PHHs (Fig. 2H). We found that miR-130a gRNA and the miR-130a inhibitor decreased miR-130a expression, by 95% ± 1% (Fig. 3A and F) and 65% ± 3% (data not shown), respectively, and increased *PKLR* mRNA expression, by 2.37 ± 0.12- and 1.87 ± 0.1-fold (Fig. 3B and G), respectively, in Huh7.5.1 cells. We also observed a moderate reduction of *PKLR* mRNA expression after JFH1 infection (Fig. 3B). As expected, we found that miR-130a knocked down increased HCV replication >2-fold in both the gRNA and miR-130a inhibitor treatment groups compared to that in the respective negative-control groups (Fig. 3C and H). miR-130a gRNA significantly increased *PKLR* and HCV core protein expression in JFH1-infected Huh7.5.1 cells (Fig. 3D). Similar findings were observed in OR6 replicon cells, which harbor a genotype 1b full-length HCV replicon with luciferase as a reporter (35, 36). The miR-130a mimic reduced and the miR-130a inhibitor and gRNA increased HCV replication in OR6 cells (data not shown). These findings indicate that miR-130a regulates HCV replication through the targeting of *PKLR* in both an HCV JFH1 infection model and an HCV OR6 replicon model.

### miR-130a regulates HBV replication through targeting of *PKLR*.

**FIG 4:**
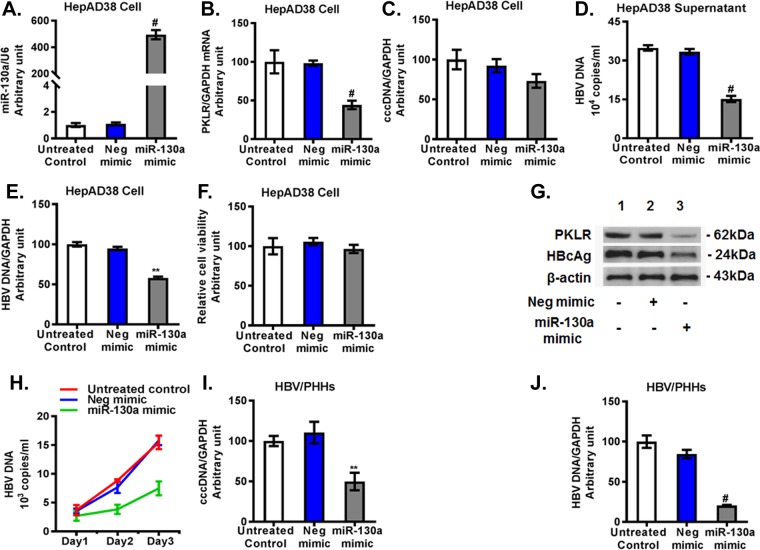
Overexpression of miR-130a inhibited *PKLR* expression and HBV replication. A negative-control mimic or miR-130a mimic was transfected into HepAD38 cells or PHHs. HBV from HepAD38 cells was used to infect cells for 72 h. miR-130a, HBV cccDNA, HBV DNA, and *PKLR* mRNA in cells, as well as HBV DNA in supernatants, were quantified using qPCR. *, *P* < 0.05; **, *P* < 0.01; #, *P* < 0.001 (for comparisons of the indicated miR-130a and negative-control miRNA treatments). (A) The miR-130a mimic increased the miR-130a level. (B) The miR-130a mimic reduced the *PKLR* mRNA level. (C) The miR-130a mimic did not significantly affect HBV cccDNA in HepAD38 cells. (D) The miR-130a mimic inhibited the HBV DNA level in the supernatant. (E) The miR-130a mimic inhibited HBV DNA in HepAD38 cells. (F) The miR-130a mimic did not significantly affect the viability of HepAD38 cells. (G) The miR-130a mimic decreased PKLR and HBcAg protein levels in HepAD38 cells. (H) The miR-130a mimic decreased the HBV DNA level in the supernatant of PHHs in a time-dependent manner. (I) The miR-130a mimic inhibited HBV cccDNA in PHHs. (J) The miR-130a mimic inhibited the total HBV DNA level in PHHs.

We found that miR-130a mimic overexpression (Fig. 4A) downregulated the *PKLR* mRNA level 56% ± 6% compared to that with the negative-control mimic treatment in HepAD38 cells (Fig. 4B). The miR-130a mimic modestly reduced the HBV covalently closed circular DNA (cccDNA) level (Fig. 4C) and significantly decreased total HBV DNA, by about 57% ± 3% in the HepAD38 cell supernatant (Fig. 4D) and by approximately 40% ± 2% in HepAD38 cells (Fig. 4E). The miR-130a mimic did not significantly affect cell viability (Fig. 4F). Western blotting confirmed that the miR-130a mimic reduced PKLR and HBcAg protein levels (Fig. 4G). We found that the HBV DNA level in the supernatant of PHHs exposed to infectious HBV increased markedly, in a time-dependent manner (Fig. 4H), confirming that PHHs support HBV infection and replication. However, the miR-130a mimic significantly lowered HBV DNA levels in the supernatants of PHHs compared to those with untreated cells or cells treated with the negative-control mimic (Fig. 4H). Moreover, we found that the miR-130a mimic decreased HBV cccDNA and total DNA levels, by 50% and 80%, respectively, in PHHs 72 h following HBV exposure (Fig. 4I and J). These findings collectively indicate that miR-130a regulates HBV replication in HepAD38 cells and PHHs.

**FIG 5:**
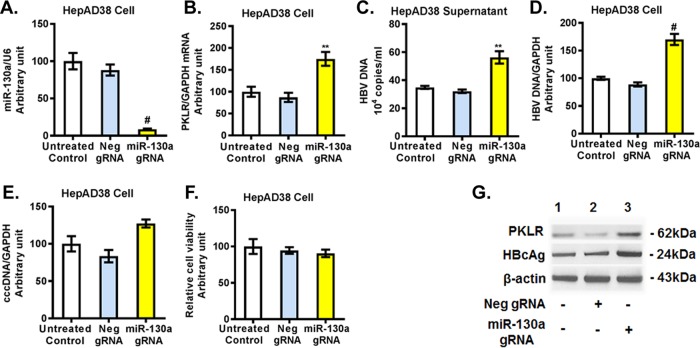
miR-130a knockdown increased *PKLR* expression and HBV replication in HepAD38 cells. miR-130a gRNA or negative-control gRNA was transfected into HepAD38 cells for 72 h. (A) miR-130a gRNA decreased the miR-130a level. (B) miR-130a gRNA increased the *PKLR* mRNA level. (C) miR-130a gRNA increased the HBV DNA level in the HepAD38 cell supernatant. (D) miR-130a gRNA increased the HBV DNA level in HepAD38 cells. (E) miR-130a gRNA did not significantly affect HBV cccDNA levels in HepAD38 cells. (F) miR-130a gRNA or negative-control gRNA did not significantly affect the viability of HepAD38 cells. (G) miR-130a gRNA increased PKLR and HBcAg protein levels.

We further investigated whether miR-130a regulates HBV replication through *PKLR*. We found that miR-130a gRNA significantly knocked down miR-130a expression (Fig. 5A) and increased the *PKLR* mRNA level (Fig. 5B) in HepAD38 cells. miR-130a gRNA significantly increased total HBV DNA in HepAD38 cell culture supernatant (Fig. 5C) and in HepAD38 cells (Fig. 5D), and it modestly increased the HBV cccDNA level (Fig. 5E) compared to that with negative-control gRNA (Fig. 5A to E). miR-130a gRNA did not significantly affect cell viability (Fig. 5F). Western blotting confirmed that miR-130a gRNA increased the PKLR and HBcAg protein levels (Fig. 5G). Interestingly, no significant change in cccDNA expression in HepAD38 cells was observed with either miR-130a mimic or miR-130a gRNA treatment (Fig. 4C and 5E). We speculate that the treatment time (72 h) may have been insufficient to alter cccDNA expression in HepAD38 cells.

### PKLR regulates HCV replication.

**FIG 6:**
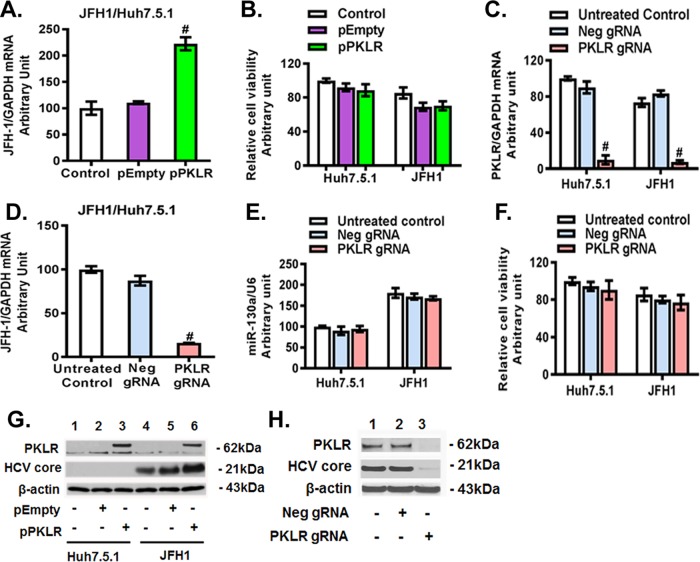
PKLR regulated HCV replication in JFH1-infected Huh7.5.1 cells. The pPKLR or pEmpty plasmid and the *PKLR* or negative-control gRNA were transfected into Huh7.5.1 cells. HCV JFH1 was inoculated into the appropriate wells and incubated for 48 h. (A) *PKLR* overexpression increased HCV replication. (B) *PKLR* overexpression did not affect cell viability. (C) *PKLR* gRNA decreased the *PKLR* mRNA level. (D) *PKLR* gRNA inhibited HCV RNA replication. (E) *PKLR* gRNA did not affect miR-130a expression. (F) *PKLR* gRNA did not affect cell viability. (G) *PKLR* overexpression increased HCV core protein levels. (H) *PKLR* gRNA decreased PKLR and HCV core protein levels.

We found that overexpression of *PKLR* had a significant stimulatory effect on HCV RNA replication (>2-fold) and HCV core protein expression compared to those with the empty vector (Fig. 6A and G). PKLR overexpression did not significantly affect the viability of Huh7.5.1 and JFH1-infected cells (Fig. 6B). In contrast, *PKLR* gRNA significantly knocked down *PKLR* mRNA (Fig. 6C) and protein (Fig. 6H) expression and reduced HCV RNA (Fig. 6D) and HCV core protein (Fig. 6H) levels. We also confirmed the inhibitory effect of *PKLR* knockdown on HCV replication by using *PKLR* siRNA (data not shown). However, we found that neither *PKLR* gRNA (Fig. 6E) nor *PKLR* siRNA (data not shown) had an effect on miR-130a expression. *PKLR* gRNA did not significantly affect the viability of Huh7.5.1 and JFH1-infected cells (Fig. 6F). Western blotting confirmed that *PKLR* overexpression increased PKLR and HCV core expression levels (Fig. 6G) and that *PKLR* gRNA decreased PKLR and HCV core protein levels (Fig. 6H). These findings indicate that *PKLR* lies downstream of miR-130a.

### PKLR regulates HBV replication.

**FIG 7:**
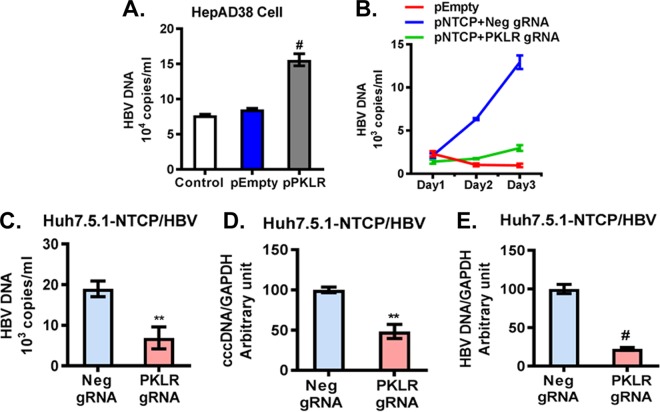
*PKLR* regulated HBV replication. (A) *PKLR* overexpression increased the HBV DNA level in HepAD38 cell supernatant. (B) HBV replication in HBV-infected NTCP-Huh7.5.1 cells increased in a time-dependent manner. *PKLR* gRNA lowered HBV DNA levels significantly compared to those with the negative-control gRNA, in a time-dependent manner. (C) *PKLR* gRNA reduced the HBV DNA level in cell culture supernatant. (D) *PKLR* gRNA reduced the HBV cccDNA level in HBV-infected NTCP-Huh7.5.1 cells. (E) *PKLR* gRNA reduced the HBV DNA level in HBV-infected NTCP-Huh7.5.1 cells.

We found that *PKLR* overexpression significantly increased HBV DNA levels in HepAD38 supernatants (Fig. 7A). Furthermore, we used Huh7.5.1 cells overexpressing NTCP to study HBV, based on previous reports (31, 37). We found that HBV DNA in the supernatant increased dramatically from day 1 (2.1 × 10^3^ ± 0.2 × 10^3^ copies/ml) to day 3 (12.9 × 10^3^ ± 0.8 × 10^3^ copies/ml), indicating the replication of HBV in NTCP-Huh7.5.1 cells (Fig. 7B). We also found that *PKLR* gRNA in NTCP-Huh7.5.1 cells significantly lowered HBV DNA in the supernatant compared to the level for the negative-control-gRNA-transfected NTCP-Huh7.5.1 cells (0.7 × 10^4^ ± 0.15 × 10^4^ copies/ml versus 1.8 × 10^4^ ± 0.2 × 10^4^ copies/ml) (Fig. 7C). Furthermore, we observed a 52% ± 9% decrease in cccDNA and a 78% ± 2% reduction in HBV DNA with *PKLR* gRNA compared to those with the negative-control gRNA in NTCP-Huh7.5.1 cells (Fig. 7D and E).

### Pyruvate plays a key role in regulation of HCV and HBV replication.

**FIG 8:**
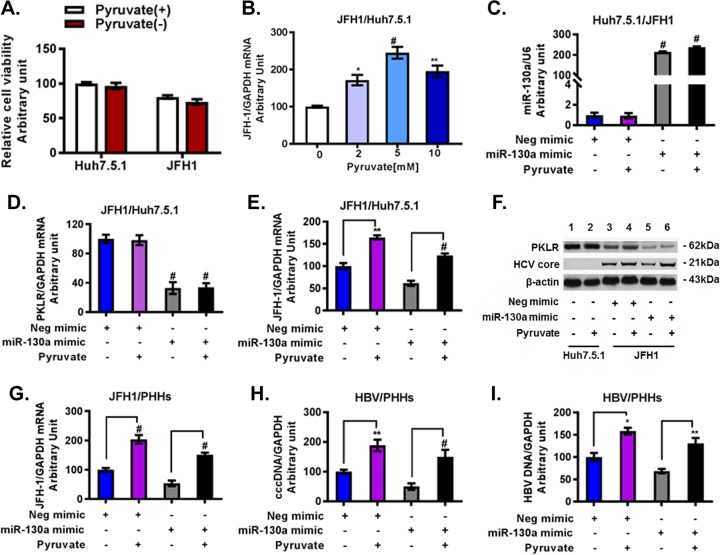
Pyruvate supplementation rescued the inhibitory effects of the miR-130a mimic on HCV and HBV replication. Huh7.5.1 cells and PHHs were cultured as described in Materials and Methods. The miR-130a mimic or negative-control mimic was transfected into Huh7.5.1 cells or PHHs. HCV JFH1 or HBV from HepAD38 cells was inoculated into the appropriate wells for HCV infection or HBV infection as described in Materials and Methods. Pyruvate was added to the appropriate wells. (A) Pyruvate did not significantly affect the viability of Huh7.5.1 cells and JFH1-infected cells. (B) Pyruvate supplementation increased HCV replication in JFH1-infected cells in a dose-dependent manner. (C) miR-130a mimic transfection significantly increased the miR-130a level in JFH1-infected cells. Supplemental pyruvate did not affect miR-130a expression. (D) The miR-130a mimic reduced the *PKLR* mRNA level in JFH1-infected cells. Supplemental pyruvate did not affect *PKLR* mRNA expression. (E) Pyruvate supplementation (5 mM) rescued the inhibitory effect of the miR-130a mimic on the HCV RNA level. (F) Pyruvate supplementation (5 mM) rescued the inhibitory effect of the miR-130a mimic on the HCV core protein level in JFH1-infected cells. (G) Pyruvate supplementation (5 mM) rescued the inhibitory effect of the miR-130a mimic on HCV replication in PHHs. (H) Pyruvate supplementation (5 mM) rescued the inhibitory effect of the miR-130a mimic on HBV cccDNA in PHHs. (I) Pyruvate supplementation (5 mM) rescued the inhibitory effect of the miR-130a mimic on HBV DNA in PHHs.

**FIG 9:**
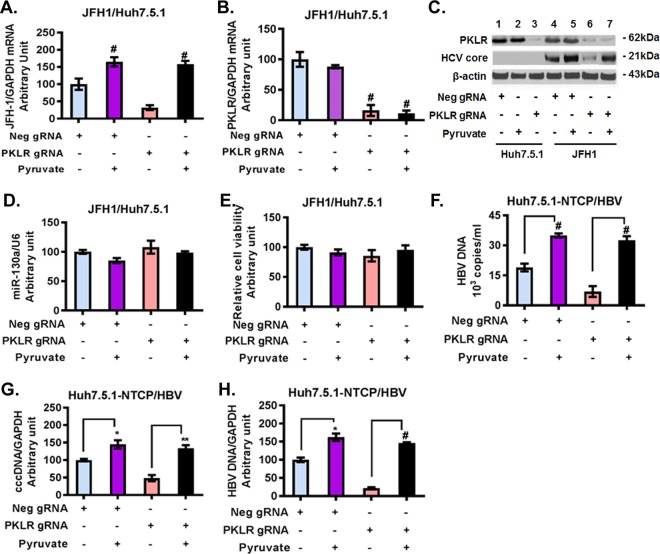
Pyruvate supplementation (5 mM) rescued the inhibitory effects of *PKLR* gRNA on HCV and HBV replication. HCV and HBV infections were performed as described in Materials and Methods. Pyruvate was added to the appropriate wells. *, *P* < 0.05; **, *P* < 0.01; #, *P* < 0.001 (for comparisons of the indicated pyruvate treatments and controls). (A) Pyruvate supplementation (5 mM) rescued the inhibitory effect of *PKLR* gRNA on HCV replication in JFH1-infected cells. (B) Pyruvate supplementation (5 mM) did not affect *PKLR* mRNA expression in JFH1-infected cells without and with pyruvate. (C) Pyruvate supplementation (5 mM) rescued the inhibitory effect of *PKLR* gRNA on the HCV core protein level in JFH1-infected cells. (D) Pyruvate supplementation (5 mM) did not affect miR-130a expression in JFH1-infected cells. (E) Pyruvate supplementation (5 mM) did not affect cell viability in JFH1-infected cells. (F) Pyruvate supplementation (5 mM) rescued the inhibitory effect of *PKLR* gRNA on the HBV DNA level in HBV-infected NTCP-Huh7.5.1 cell culture supernatant. (G) Pyruvate supplementation (5 mM) rescued the inhibitory effect of *PKLR* gRNA on HBV cccDNA replication in HBV-infected NTCP-Huh7.5.1 cells. (H) Pyruvate supplementation (5 mM) rescued the inhibitory effect of *PKLR* gRNA on HBV DNA replication in HBV-infected NTCP-Huh7.5.1 cells.

We next investigated whether the regulatory effect of miR-130a on HCV and HBV production is mediated through the enzymatic reaction controlled by *PKLR*, which results in pyruvate production. The effects of HCV JFH1 infection were tested in Huh7.5.1 cells in Dulbecco's modified Eagle's medium (DMEM), with or without the addition of sodium pyruvate to the medium. We found that HCV JFH1 infection modestly reduced cell viability compared to that of uninfected Huh7.5.1 cells. However, there was no significant difference in cell viability between Huh7.5.1 cells and JFH1-infected Huh7.5.1 cells cultured in DMEM with pyruvate and without pyruvate (Fig. 8A). Moreover, pyruvate supplementation increased HCV RNA in a dose-dependent manner (Fig. 8B). Higher concentrations of pyruvate (2 mM and 5 mM) significantly increased HCV RNA levels compared to those with 0 mM pyruvate in JFH1-infected Huh7.5.1 cells; however, an even higher concentration of pyruvate (10 mM) did not further enhance HCV RNA levels (Fig. 8B). We therefore selected 5 mM pyruvate to further investigate the effects of pyruvate on HCV or HBV replication (Fig. 8 and 9). We confirmed that the miR-130a mimic (overexpression) decreased *PKLR* and HCV RNA and protein expression levels (Fig. 8C to F). Supplemental pyruvate (5 mM) rescued the inhibitory effect of the miR-130a mimic on HCV RNA replication reduction in JFH1-infected Huh7.5.1 cells (Fig. 8E). Western blotting confirmed that miR-130a decreased the PKLR protein level and that pyruvate rescued HCV core protein expression (Fig. 8F). The miR-130a mimic reduced HCV RNA (Fig. 8G) and HBV DNA (Fig. 8H and I) replication levels in PHHs. Supplemental pyruvate rescued HCV RNA or HBV DNA replication in PHHs (Fig. 8G to I). We found that *PKLR* gRNA decreased HCV RNA and HCV core protein expression in JFH1-infected Huh7.5.1 cells (Fig. 9A to C). Supplemental pyruvate also rescued the *PKLR* gRNA inhibition of HCV replication (Fig. 9A and C). We confirmed that *PKLR* gRNA knocked down *PKLR* mRNA and protein expression (Fig. 9B and C). In addition, miR-130a expression and cell viability were not significantly affected by *PKLR* gRNA or supplemental pyruvate (Fig. 9D and E). We further examined *PKLR* gRNA and pyruvate effects on HBV replication. We found that *PKLR* gRNA reduced HBV DNA levels in the supernatant and in NTCP-Huh7.5.1 cells (Fig. 9F to H). Supplemental pyruvate rescued the *PKLR* gRNA inhibition of HBV replication (Fig. 9F to H). These findings indicate that PKLR is a critical protein for both HCV and HBV replication.

## DISCUSSION

Pyruvate occupies a critical node in central carbon metabolism, and alterations in pyruvate metabolism can cause human disease (20). Pyruvate is the end product of glycolysis; it is generated from PEP and is predominantly converted into fatty acid via acetyl-coenzyme A (acetyl-CoA) in the liver (38). The conversion of PEP to pyruvate is catalyzed by pyruvate kinase. There are four tissue-specific isozymes: L, R, M1, and M2. The PKL and PKR transcripts are encoded by the same gene (*PKLR*). PKR is found only in erythrocytes, whereas PKL is expressed in the liver, kidney, and small intestines (39). The PKL isozyme is regulated at the transcriptional and posttranscriptional levels in response to various hormones.

Regulation of pyruvate metabolism also plays a vital role in viral infection, as viruses rely on host energy and biosynthetic pathways for their replication and proliferation. It has been shown that HCV and HBV infections are accompanied by host cellular metabolic reprogramming (40, 41). Previous studies reported reductions in *PKLR* expression and pyruvate secretion in chronic HCV infections (40) as well as in HBV replicon HepG2.2.15 cells (41). There are several reports of decreased *PK* expression in dengue virus-infected Huh-7 cells (42) and human cytomegalovirus (HCMV)-infected fibroblasts (43). However, host cells are able to sense the viral infection and to generate immune responses to combat and contain the invading virus. This reaction is energy intensive and is regulated by the host metabolism, including glucose and lipid metabolism.

The regulation of metabolism and antiviral protection strategies by miRNAs has mostly been investigated independently. miR-130a has been shown to regulate insulin sensitivity through targeting of GRB10 (44). Insulin is a key hormone that is associated with glucose metabolism and can regulate PKL expression (45). It has been reported that interaction between miR-122 and HCV reduces miR-122 bioavailability (14). In this study, we confirmed the regulatory effect of miR-130a on HCV and HBV. We identified *PKLR* as a direct target gene of miR-130a. miR-130a regulated HCV and HBV replication through targeting of *PKLR*. It has previously been demonstrated that overexpression of miR-130a inhibits HBV DNA replication through targeting of *PGC1α* and *PPARγ* (13). *PKLR* was also found to be the target gene of miR-338-3p (46). miR-338-3p suppressed the Warburg effects of hepatocellular carcinoma (HCC) cells by targeting *PKLR* (46), indicating that miR-130a likely plays a role in HCC development. We further discovered the regulatory effect of PKLR and its product pyruvate on HCV and HBV replication. *PKLR* overexpression or supplementation with pyruvate increased both HCV and HBV replication in several infection models. In contrast, knockdown of *PKLR* expression reduced HCV and HBV replication, and supplementation with pyruvate rescued the inhibitory effects of the miR-130a mimic and *PKLR* gRNA on virus infection.

Other viral infections depend on host cellular metabolism, including glycolysis, to support viral genome replication and virion assembly (47). For example, pyruvate has been identified as a host cellular protein within influenza virus particles (48). Influenza virus replication also induces host pyruvate kinase M production (49). HCV infection induces glycolysis through an increase in glycolytic enzymes in Huh7 cells (40). HCV NS5A protein interaction with HK2 induces an enhancement of glucose uptake and lactic acid production (50). Dengue virus requires glycolysis production for optimal replication (51). We speculate that decreasing PKLR and pyruvate levels, as well as subsequent glycolysis levels, inhibit HCV and HBV replication through reduced production of ATP and other glycolytic intermediates. Pyruvate was also found to prevent the death of cells latently infected with Kaposi's sarcoma-associated herpesvirus (KSHV) (52).

Our study identifies PKLR and pyruvate as essential metabolic components for HCV and HBV replication. It further raises the possibility that inhibition of PKLR and pyruvate may be useful as a short-term measure to restrict HCV or HBV infection. It has been reported that the interferon (IFN)-induced transmembrane protein IFITM1 is another potential miR-130a target (34). HCV infection is associated with increased miR-130a expression in human liver biopsy specimens and in an HCV infection cell culture model, and IFITM1 overexpression inhibits HCV replication (34). We found that miR-130a gRNA blocked IFN-stimulated response element (ISRE)-directed signaling (data not shown). However, *PKLR* gRNA did not affect ISRE signaling (data not shown). Our findings indicate that miR-130a regulation of the IFN-induced ISRE pathway and IFN-stimulated gene (ISG) innate immunity is independent of *PKLR*.

Our study systematically investigated the association of the PKLR/pyruvate enzymatic reaction and HCV/HBV replication, which is regulated by the small noncoding RNA miR-130a. We therefore propose a miR-130a regulation pathway model in which miR-130a regulates HCV and HBV replication through *PKLR* expression and subsequent pyruvate metabolism. Further understanding of the mechanisms by which miRNA, PKLR, and pyruvate regulate HCV and HBV replication through ATP and other glycolytic intermediates may facilitate the development of strategies to prevent establishment of persistent infection for HCV, HBV, and possibly other viral infections.

## MATERIALS AND METHODS

### Cell cultures and infectious viruses.

Cells of the human hepatoma cell lines Huh7.5.1 and HepAD38 (HBV production is under the control of a tetracycline-regulated promoter) were grown in DMEM with 10% fetal bovine serum (FBS). Huh7.5.1 cells were infected with the genotype 2a strain HCV JFH1 (multiplicity of infection [MOI] = 0.2) (JFH1-infected cells) as previously described (53, 54). The human hepatoma cell line OR6 (35), which harbors full-length genotype 1b HCV RNA and coexpresses Renilla luciferase, was grown in DMEM supplemented with 10% FBS and 500 μg/ml of G418 (Promega, Madison, WI). Primary human hepatocytes (PHHs) were purchased from Triangle Research Labs (NC, USA) and cultured according to the manufacturer's protocol. To reconstitute NTCP expression, Huh7.5.1 cells were transfected with an NTCP plasmid by use of Lipofectamine LTX (Life Technologies) for 48 h. The expression levels of NTCP in the candidate cell clones were measured by Western blotting. Infectious HBV was derived from the culture supernatant of HepAD38 cells, and infections of PHHs and NTCP-Huh7.5.1 cells were carried out as previously described (31, 37, 55, 56). In brief, NTCP-Huh7.5.1 cells or PHHs were incubated with HBV inoculum for 24 h. The medium was aspirated, the cells were washed three times with phosphate-buffered saline (PBS), and then the medium was replaced with fresh medium. HBV DNA in the supernatant or in cells was isolated using a QIAamp DNA minikit (Qiagen, MD, USA). HBV DNA was quantified by real-time PCR.

### miR-130a mimic, miR-130a inhibitor, siRNAs, CRISPR/Cas9 gRNAs, plasmids, and transfection.

A miR-130a mimic, a miR-130a hairpin inhibitor, and the corresponding negative controls were purchased from GE Dharmacon (Lafayette, CO, USA). GenCRISPR gRNAs for miR-130a and *PKLR* were purchased from GenScript USA Inc. (Piscataway, NJ, USA). Protospacer sequences for CRISPR/Cas9 targeting of miR-130a or *PKLR* were designed by use of CRISPR Design (http://crispr.mit.edu/). The generated gRNA sequence was cloned into the vector pGS-gRNA-Neo. After transient transfection of CRISPR/Cas9 gRNA into cells by use of Lipofectamine LTX reagent (Life Technologies), G418 (Life Technologies) was added for selection of miR-130a or *PKLR* knockdown cells. *PKLR* siRNA and negative-control siRNA were purchased from GE Dharmacon and transfected into cells by use of Lipofectamine RNAiMax reagent (Thermo Fisher Scientific). pNTCP, pPKLR, and pEmpty were also transfected into cells by use of Lipofectamine LTX.

### Luciferase assay.

The bioinformatics analysis for identification of target genes of miR-130a was performed using the miRanda, RNAhybrid, TargetScan, and PITA software tools (57). Possible target genes were selected according to a flow diagram (data not shown). To verify whether the three possible target genes (*PKLR*, *IL18BP*, and *LDLR*) were direct target genes of miR-130a, a dual-luciferase reporter assay was performed. The PKLR-3′UTR-WT, PKLR-3′UTR-Mut, IL18BP-3′UTR-WT, and LDLR-3′UTR-WT plasmids were purchased from GeneCopoeia (Rockville, MD). The plasmids were constructed by cloning the 3′ UTRs of *PKLR*, *IL18BP*, and *LDLR* into the dual-luciferase vector pEZX-MT06, downstream of the firefly luciferase reporter (*hLuc*) gene. The mutant *PKLR* reporter plasmid PKLR-3′UTR-Mut was constructed by deleting the binding sites of miR-130a in the 3′ UTR of *PKLR*. The vector has a Renilla luciferase gene (*Rluc*) as an internal control. For the luciferase reporter assay, Huh7.5.1 cells (1.5 × 10^4^ cells/per well) were seeded into 96-well plates and incubated overnight. Cells were cotransfected with 50 nM miR-130a mimic (GE Dharmacon, USA) or 50 nM negative control (GE Dharmacon) and 0.1 μg/well plasmid by use of Lipofectamine LTX (Thermo, USA). Firefly and Renilla luciferase activities were measured by use of a Promega dual-luciferase reporter assay at 24 h posttransfection. The firefly luciferase/Renilla luciferase activity ratio was calculated to determine the binding between the cloned 3′ UTR and miR-130a. HCV replication in OR6 cells was determined by monitoring Renilla luciferase activity (Promega, Madison, WI). Cell viability was assessed by use of a Cell Titer-Glo luminescent cell viability assay kit (Promega, Madison, WI) according to the manufacturer's protocol.

### Pyruvate treatment.

For pyruvate treatment studies, the medium from cells grown in 10% FBS-DMEM containing pyruvate (Corning) was aspirated, washed with PBS three times, and replaced with 10% FBS-DMEM without sodium pyruvate (Corning). Supplemental sodium pyruvate (Thermo Fisher) was added at specific concentrations to the appropriate wells.

### DNA and RNA isolation and quantification.

HBV DNA was isolated from cells or supernatants by use of a QIAamp minikit. Total RNA was isolated by use of a QIA Shredder and RNeasy kit (Qiagen, Valencia, CA). Reverse transcription was performed by use of a high-capacity cDNA reverse transcription kit with random primers (Applied Biosystems Inc., Foster City, CA, USA). To quantify gene expression, quantitative real-time PCR was performed using an ABI QuantStudio 3 system (Applied Biosystems Inc.) and Power Up SYBR green master mix (Thermo Fisher Scientific, Waltham, MA). The primers used were described previously (22, 31). The reactions were performed under the following conditions: 95°C for 3 min followed by 45 cycles of 94°C for 20 s, 60°C for 30 s, and 72°C for 20 s. The mRNA level of each gene was normalized to that for glyceraldehyde-3-phosphate dehydrogenase (GAPDH) to obtain the number of mRNA arbitrary units (fold change).

### Protein sample preparation and Western blot analysis.

Cells were washed with PBS and lysed by use of RIPA buffer containing a protease inhibitor cocktail (Sigma Life Science and Biochemicals, St. Louis, MO). For HBV protein preparation, samples were heated at 56°C for 30 min. We loaded equal quantities of protein (20 μg) in all lanes. Protein samples were separated by SDS-PAGE with NuPAGE Novex precast 4 to 12% Bis-Tris gradient gels (Invitrogen, Carlsbad, CA) and blotted onto nitrocellulose membranes. The membranes were blocked with 5% bovine serum albumin (BSA) in Tris-buffered saline with Tween 20 (TBST). Primary antibodies included mouse anti-human PKLR (Santa Cruz Biotechnology, TX), mouse anti-HCV core (Fisher Scientific, Pittsburgh, PA), mouse anti-HBcAg (Abcam, Cambridge, MA), and mouse anti-β-actin (Sigma, St. Louis, MO). The secondary antibodies were horseradish peroxidase (HRP)-conjugated enhanced chemiluminescence (ECL) donkey anti-rabbit IgG and HRP-conjugated ECL sheep anti-mouse IgG (GE Healthcare Biosciences, Pittsburgh, PA). The blots were subjected to chemiluminescence assay by use of an Amersham ECL Western blotting detection kit (GE Healthcare Biosciences, Pittsburgh, PA).

### Data analysis.

Data are expressed as means ± standard deviations (SD) for at least three independent experiments unless otherwise stated. Data analysis was performed using 2-tailed Student's *t* test.
